# Supplementary material for: Cardiac Magnetic Resonance Imaging and Transthoracic Echocardiography: Investigation of Concordance between the Two Methods for Measurement of the Cardiac Chamber
Source: Medicina (Kaunas). 2019 Jun 9;55(6):260. doi: 10.3390/medicina55060260 (PMC6631713; doi:10.3390/medicina55060260)
Supplement: Supplementary file 1 [file medicina-55-00260-s001.pdf]

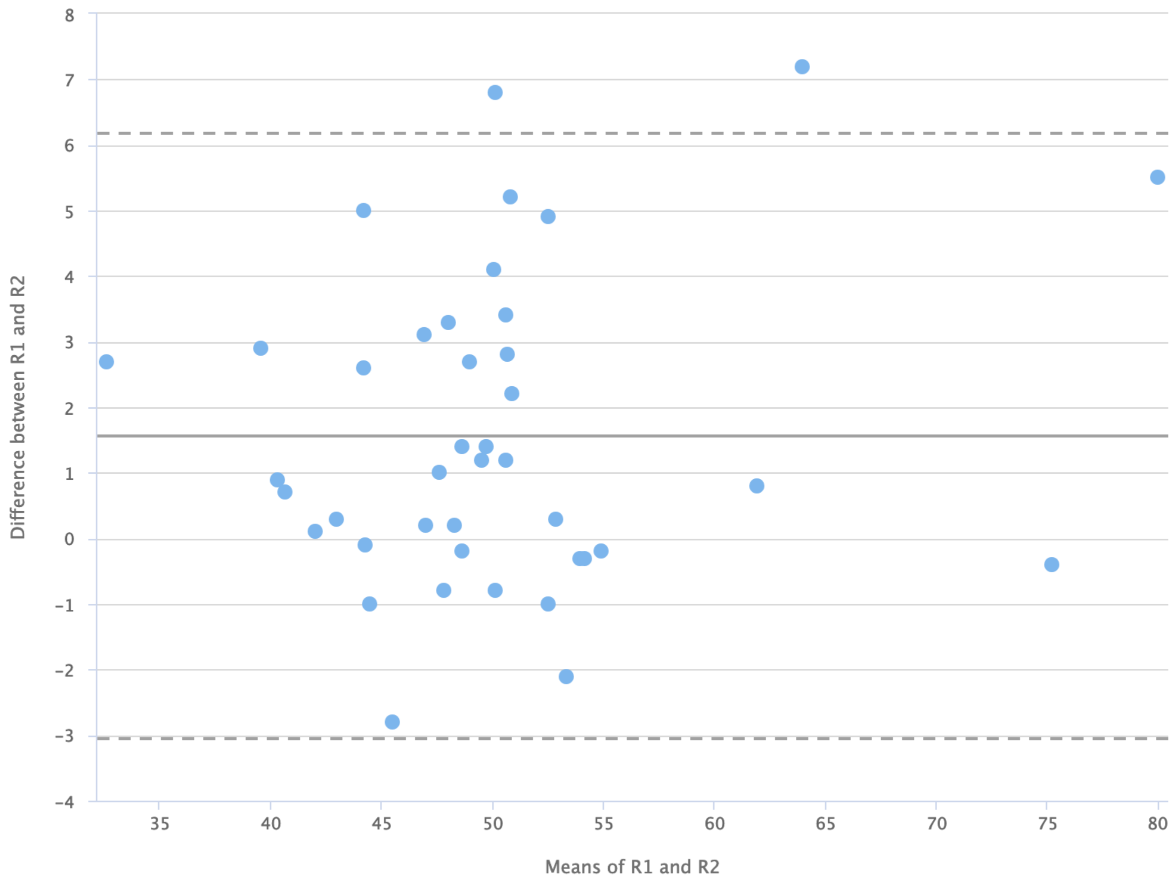

Figure S1. Bland Altman plot for the agreement between radiologist 1 and radiologist 2 for LVEDD.

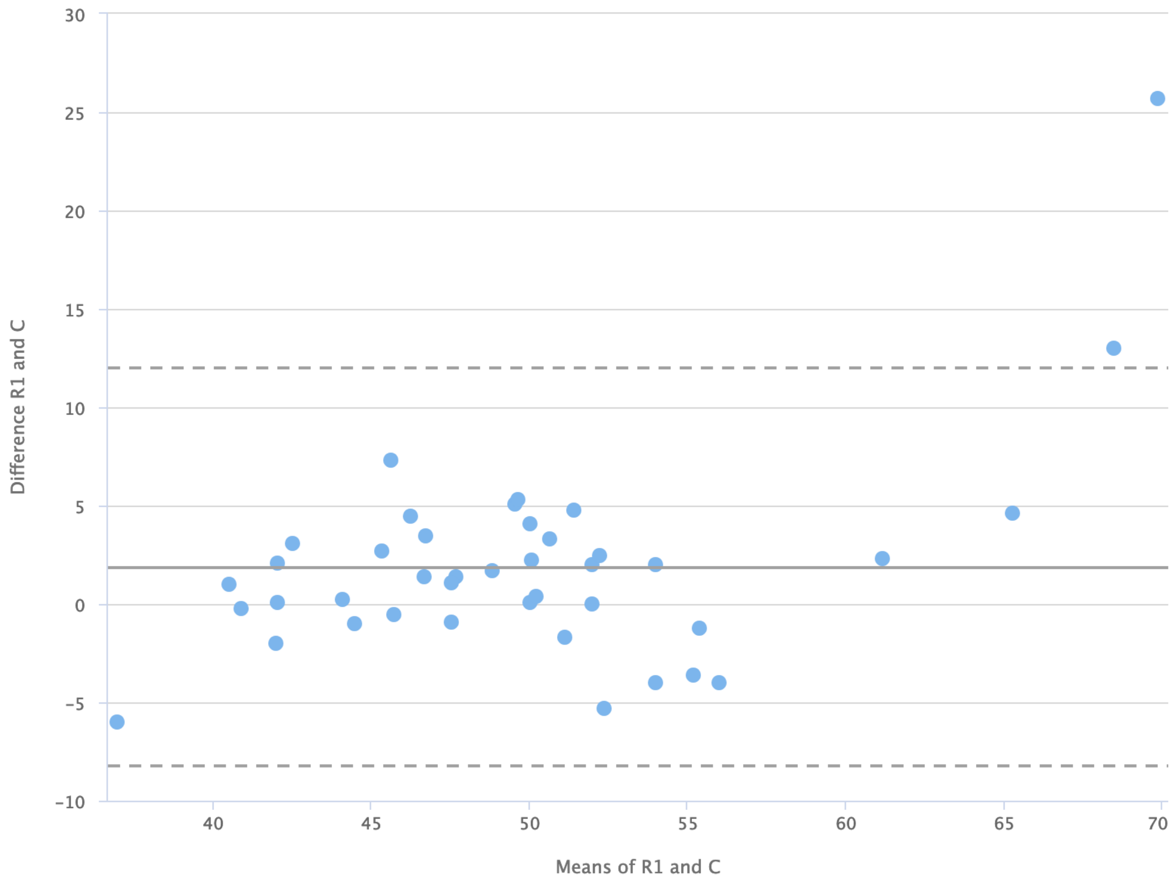

Figure S2. Bland Altman plot for the agreement between radiologist 1 and cardiologist for LVEDD.

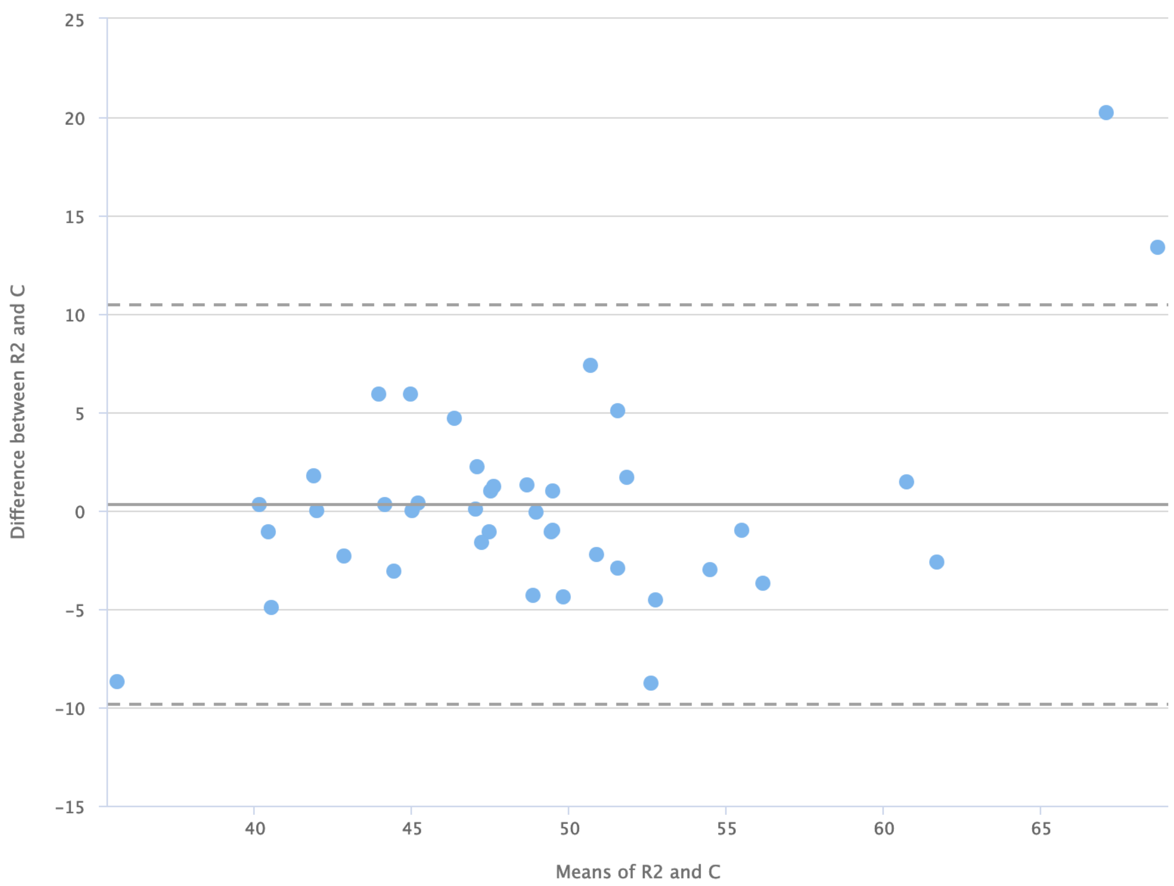

Figure S3. Bland Altman plot for the agreement between radiologist 2 and cardiologist for LVEDD.

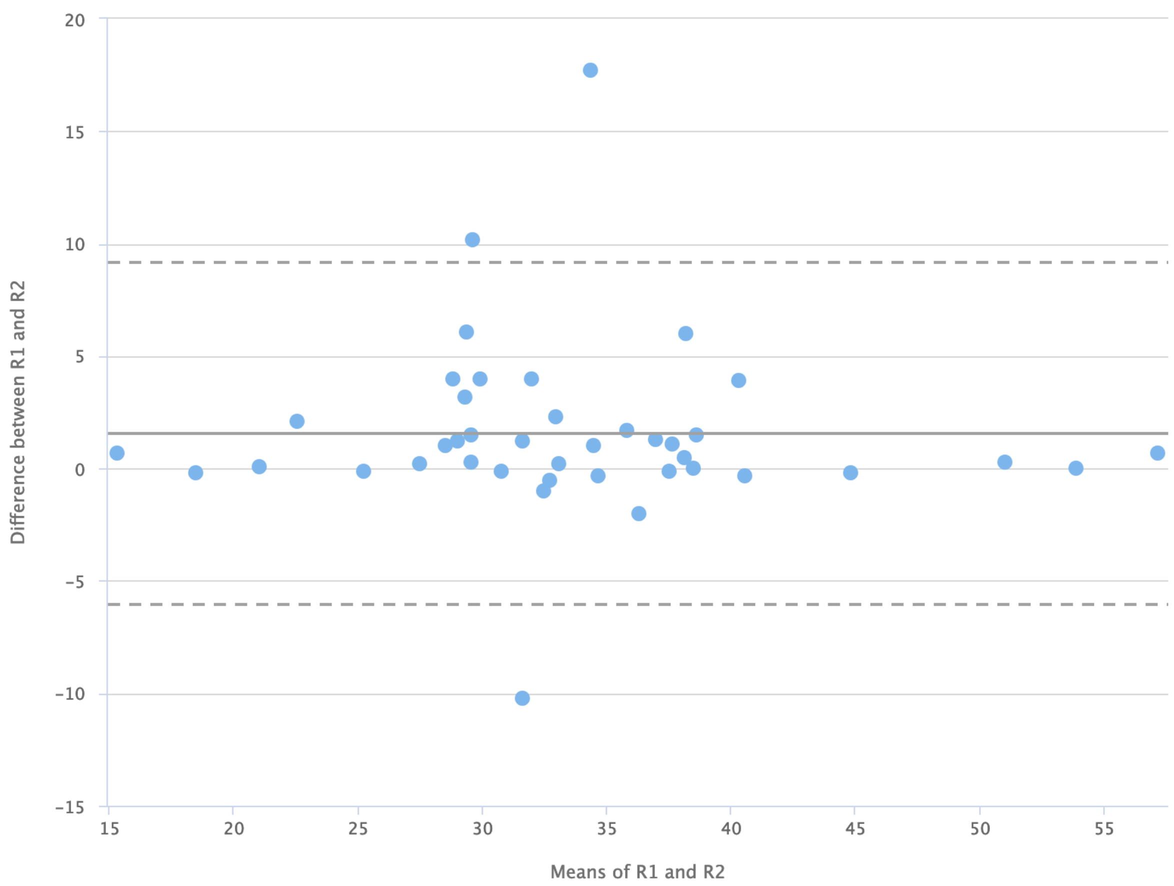

Figure S4. Bland Altman plot for the agreement between radiologist 1 and radiologist 2 for LVES.

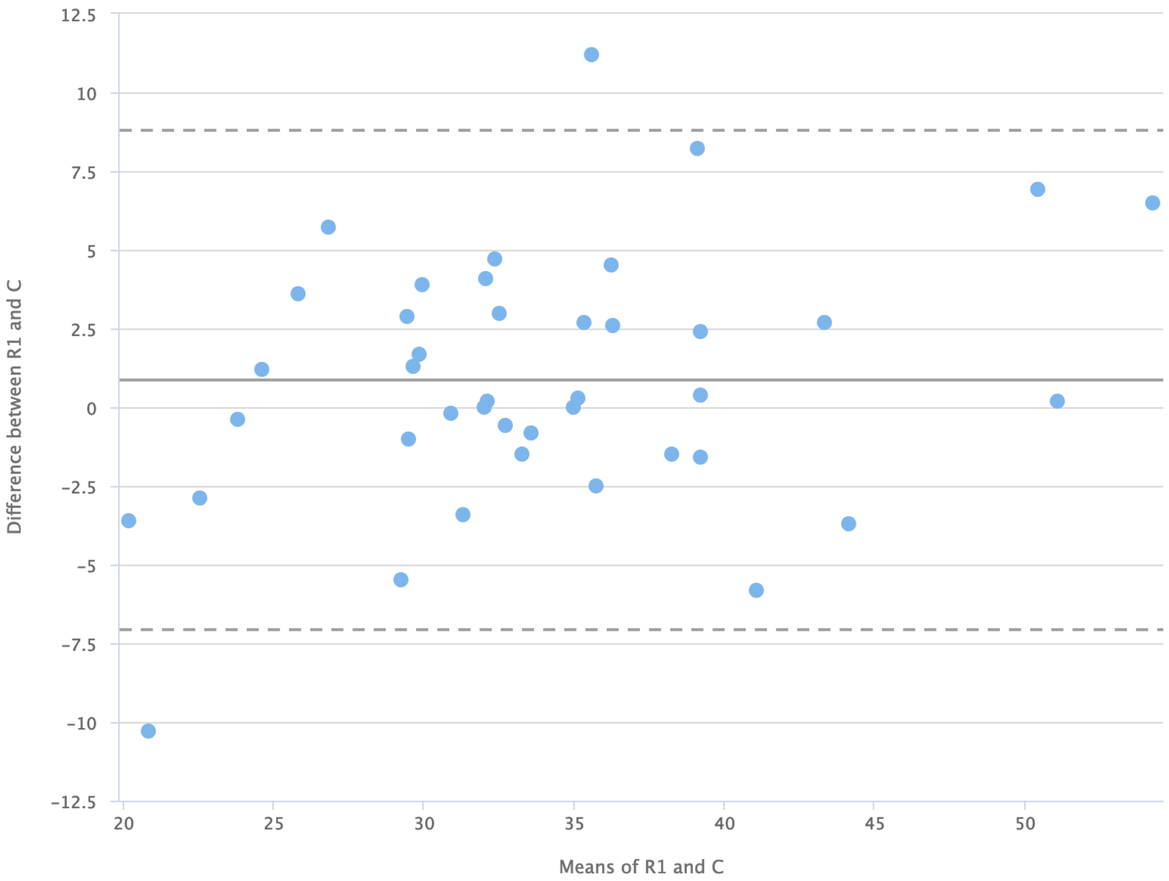

Figure S5. Bland Altman plot for the agreement between radiologist 1 and cardiologist for LVES.

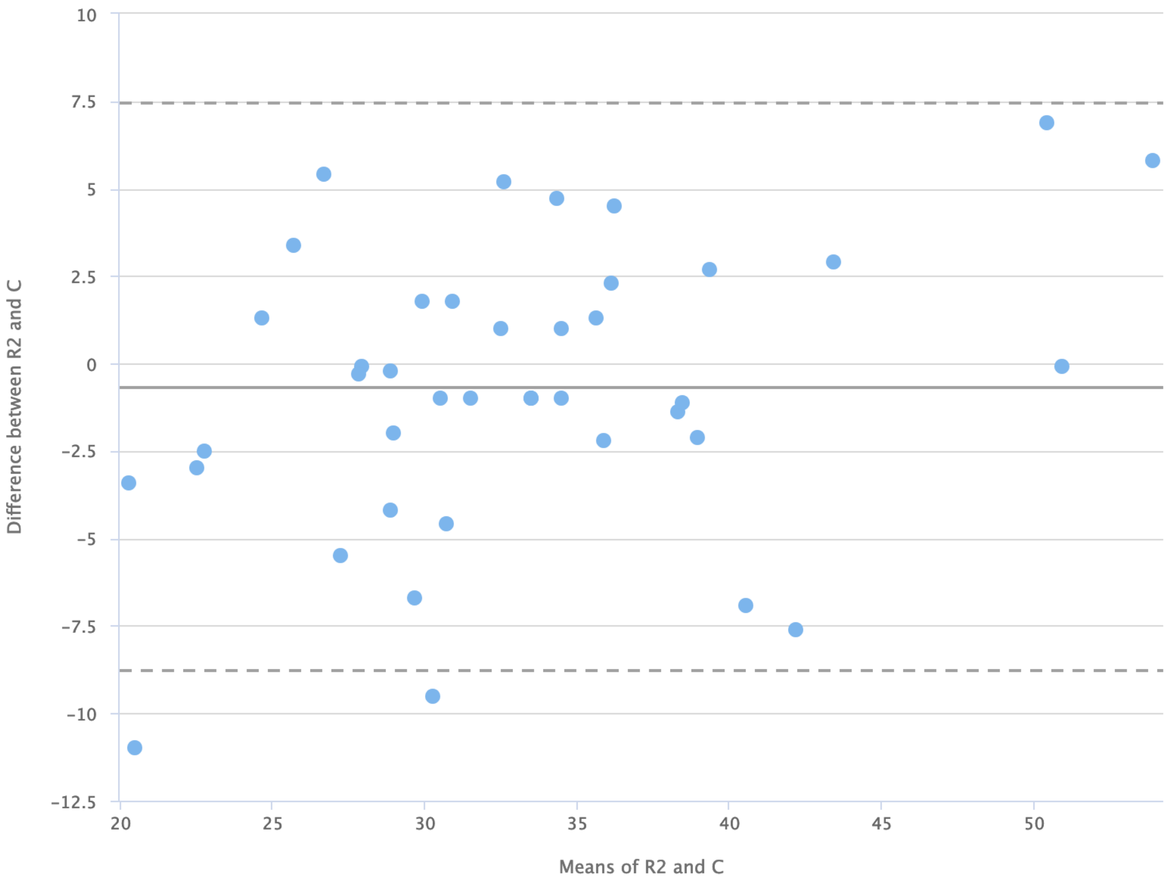

Figure S6. Bland Altman plot for the agreement between radiologist 2 and cardiologist for LVES.

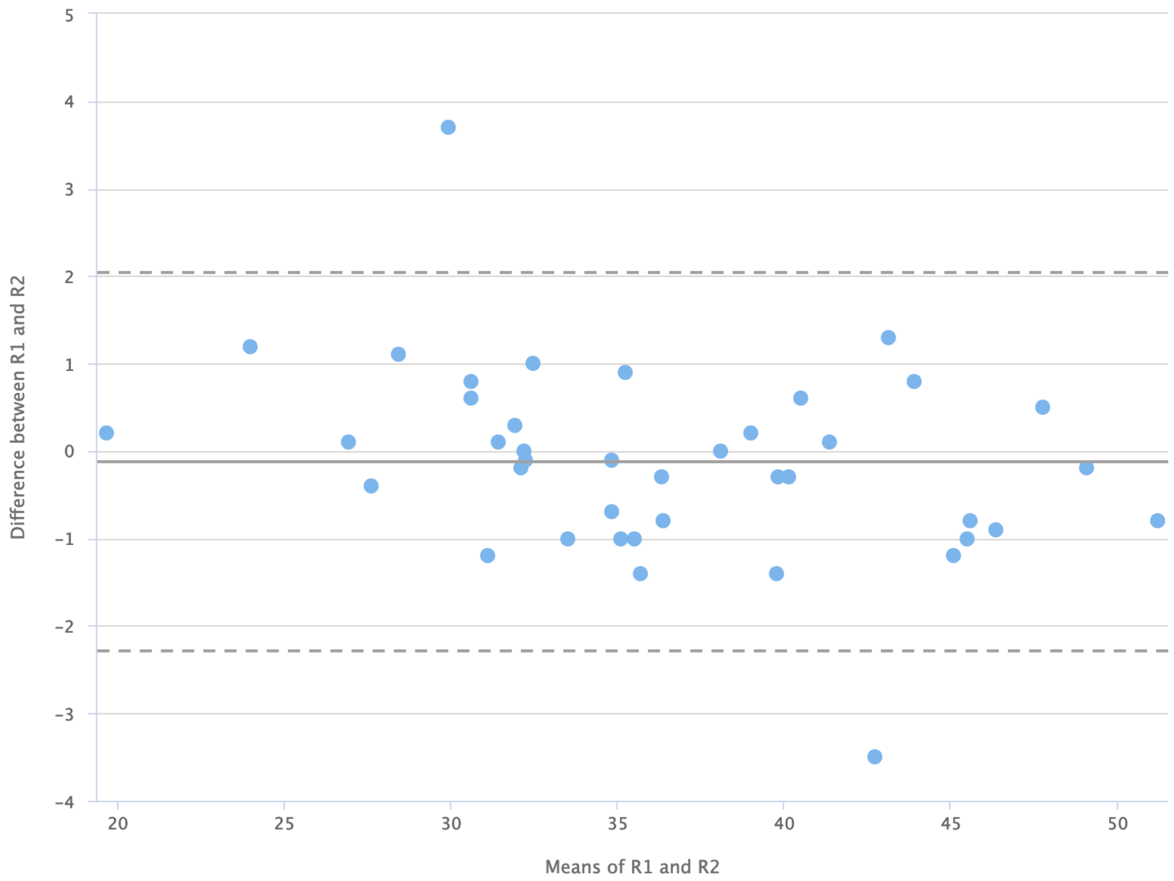

Figure S7. Bland Altman plot for the agreement between radiologist 1 and radiologist 2 for LAAP.

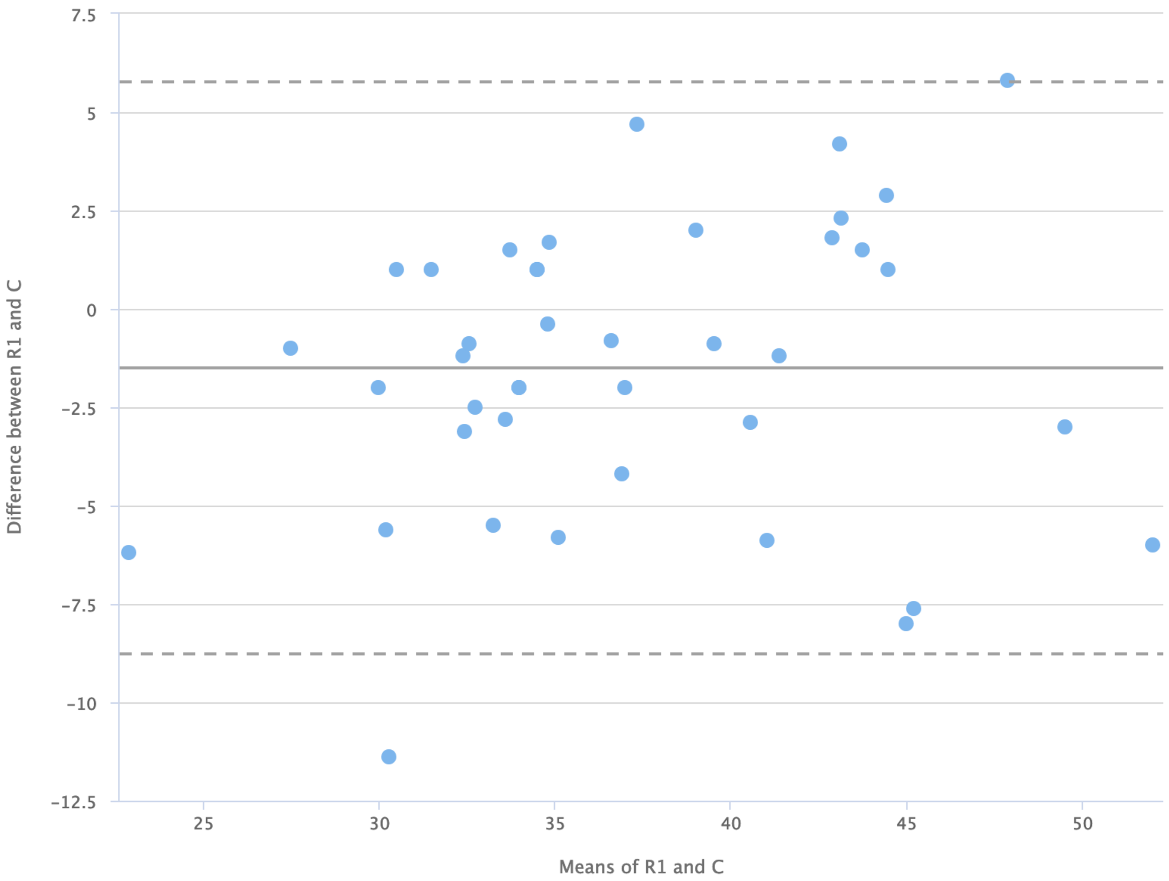

Figure S8. Bland Altman plot for the agreement between radiologist 1 and cardiologist for LAAP.

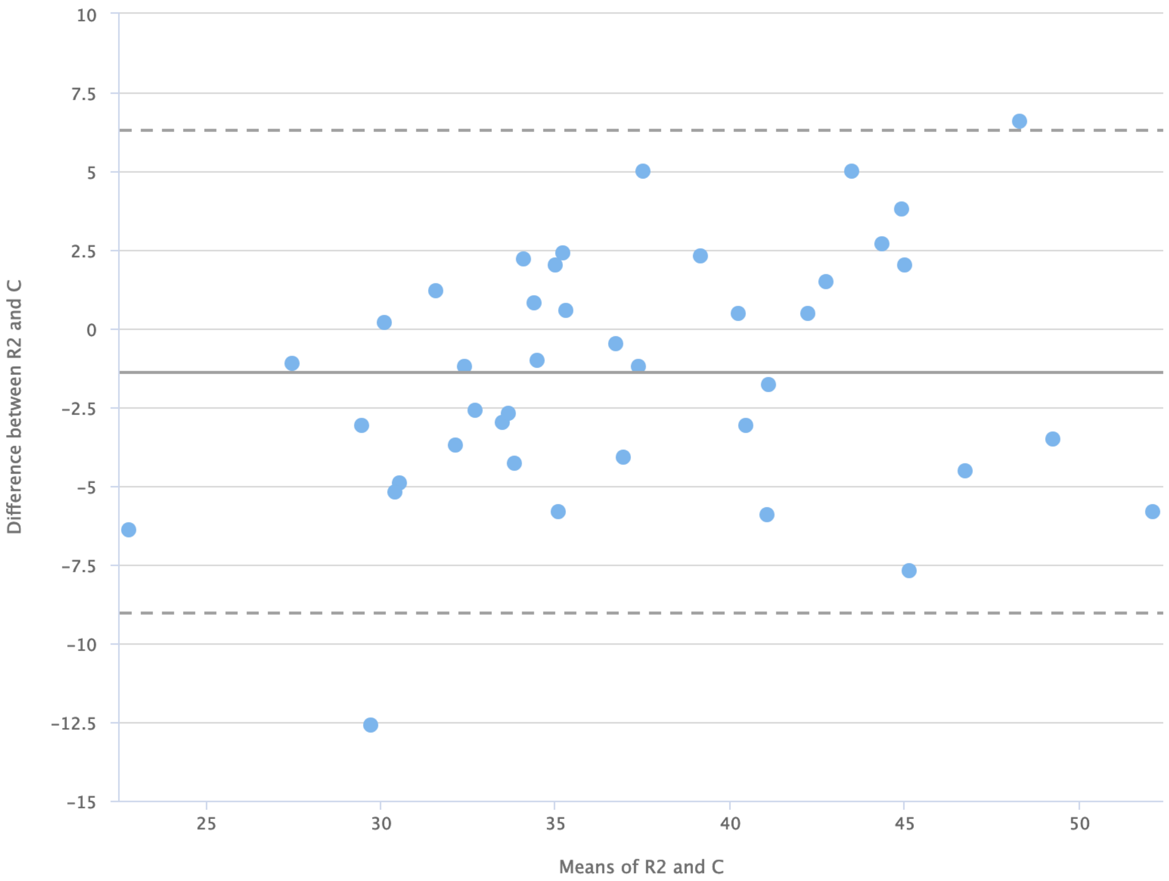

Figure S9. Bland Altman plot for the agreement between radiologist 2 and cardiologist for LAAP.

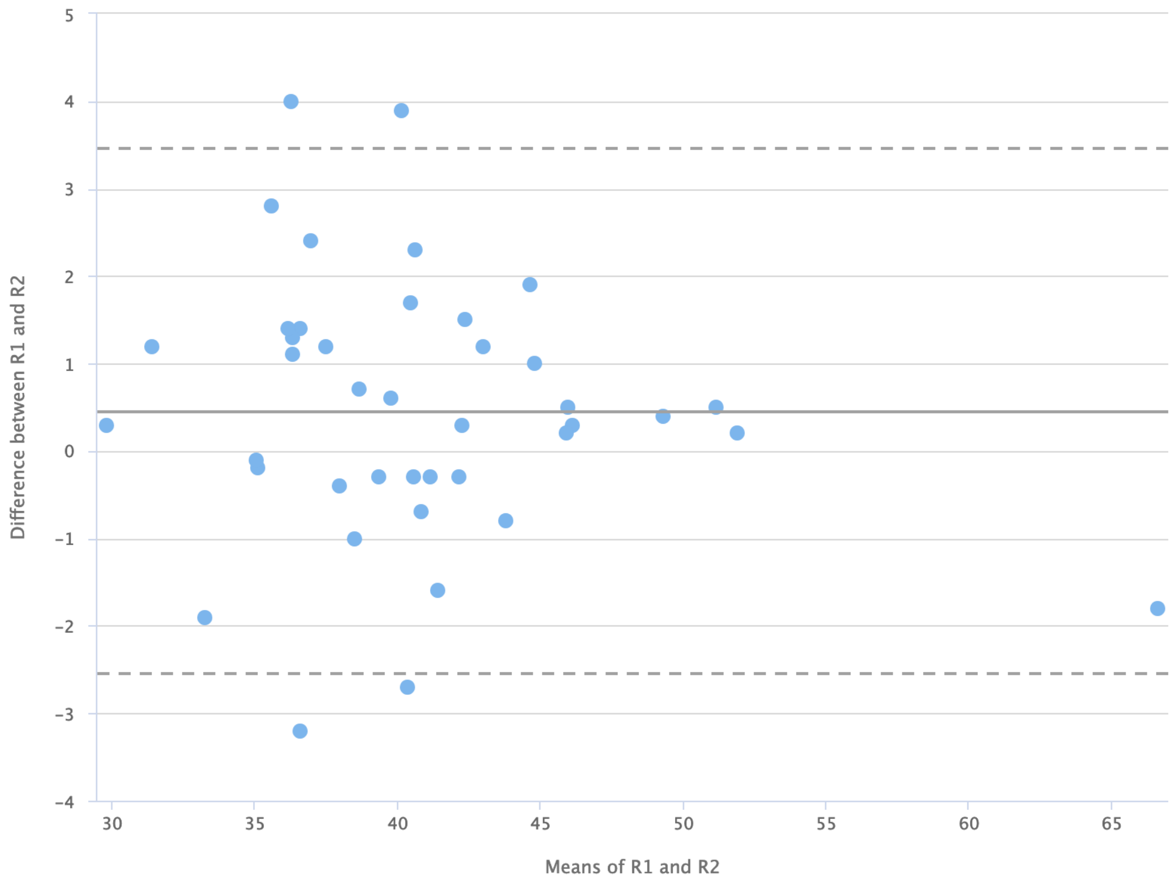

Figure S10. Bland Altman plot for the agreement between radiologist 1 and radiologist 2 for RV.

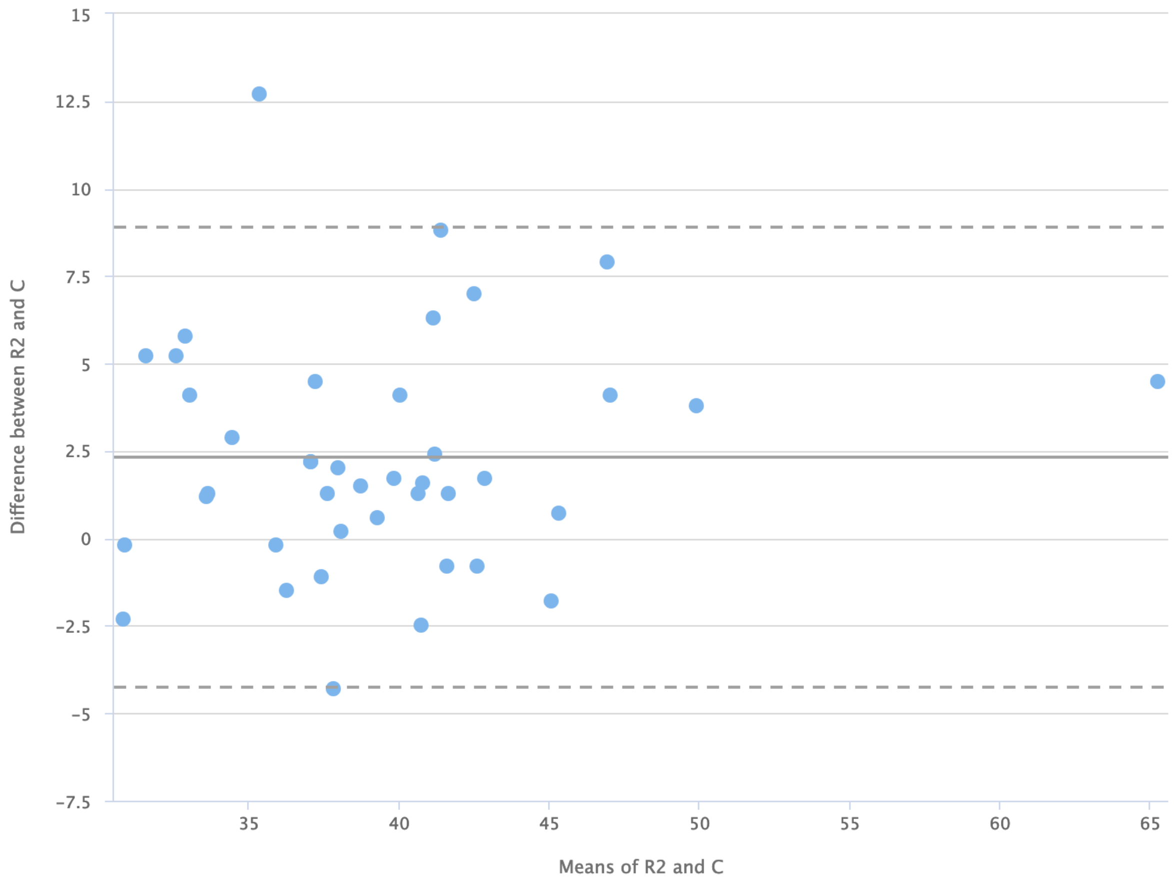

Figure S11. Bland Altman plot for the agreement between radiologist 1 and cardiologist for RV.

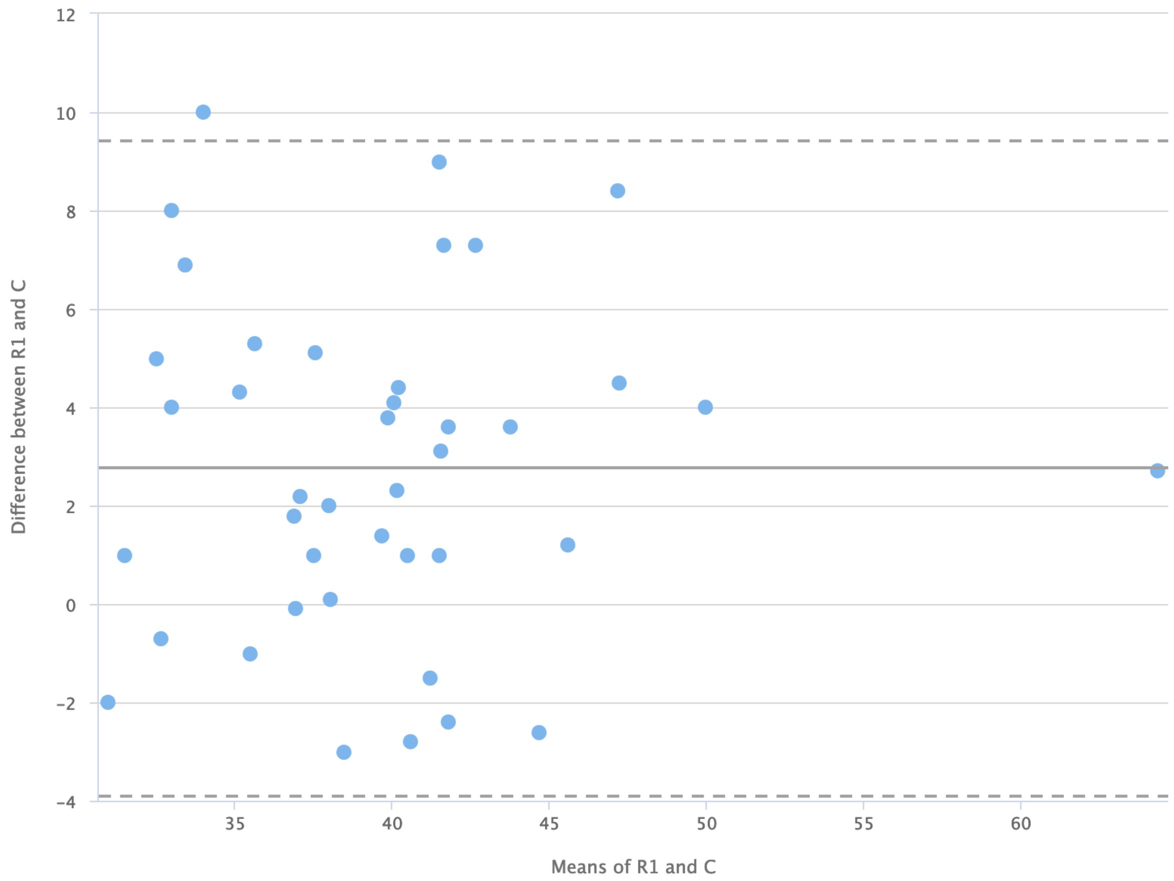

Figure S12. Bland Altman plot for the agreement between radiologist 2 and cardiologist for RV.

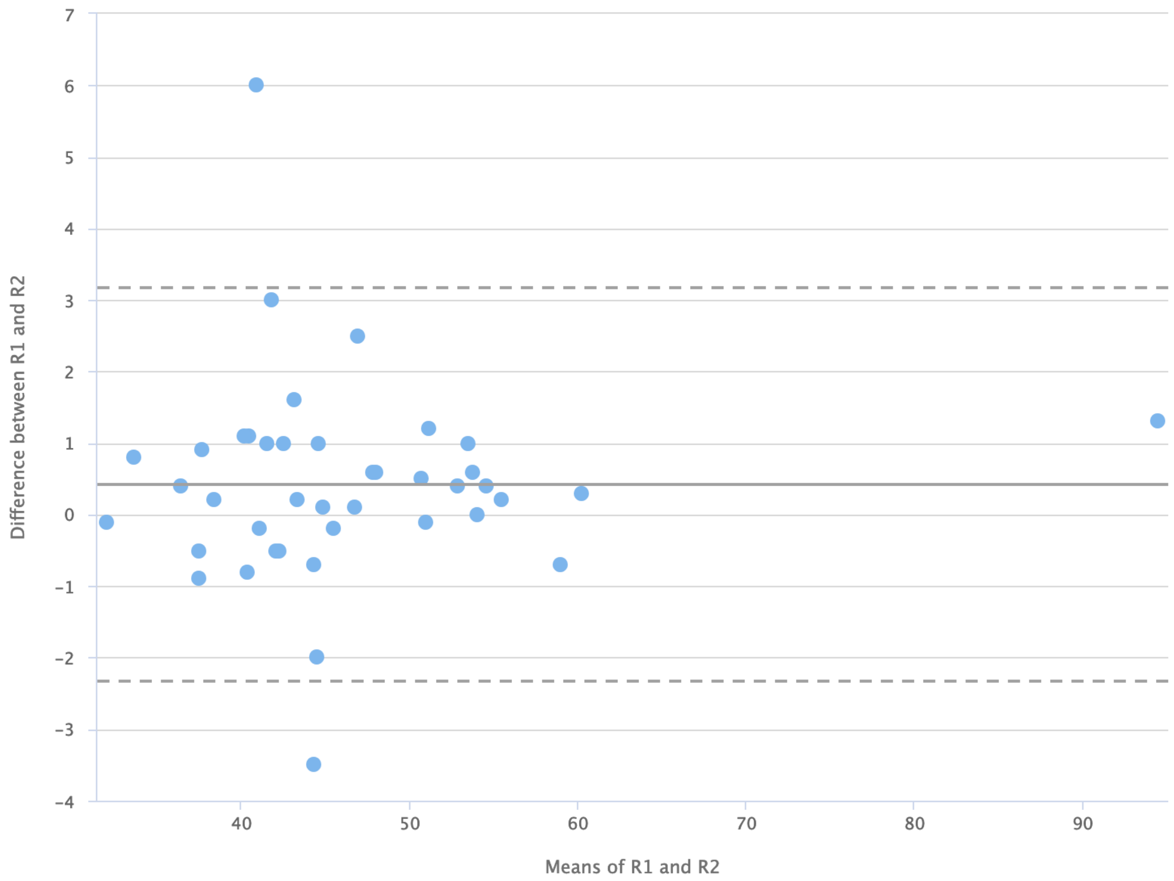

Figure S13. Bland Altman plot for the agreement between radiologist 1 and radiologist 2 for RA.

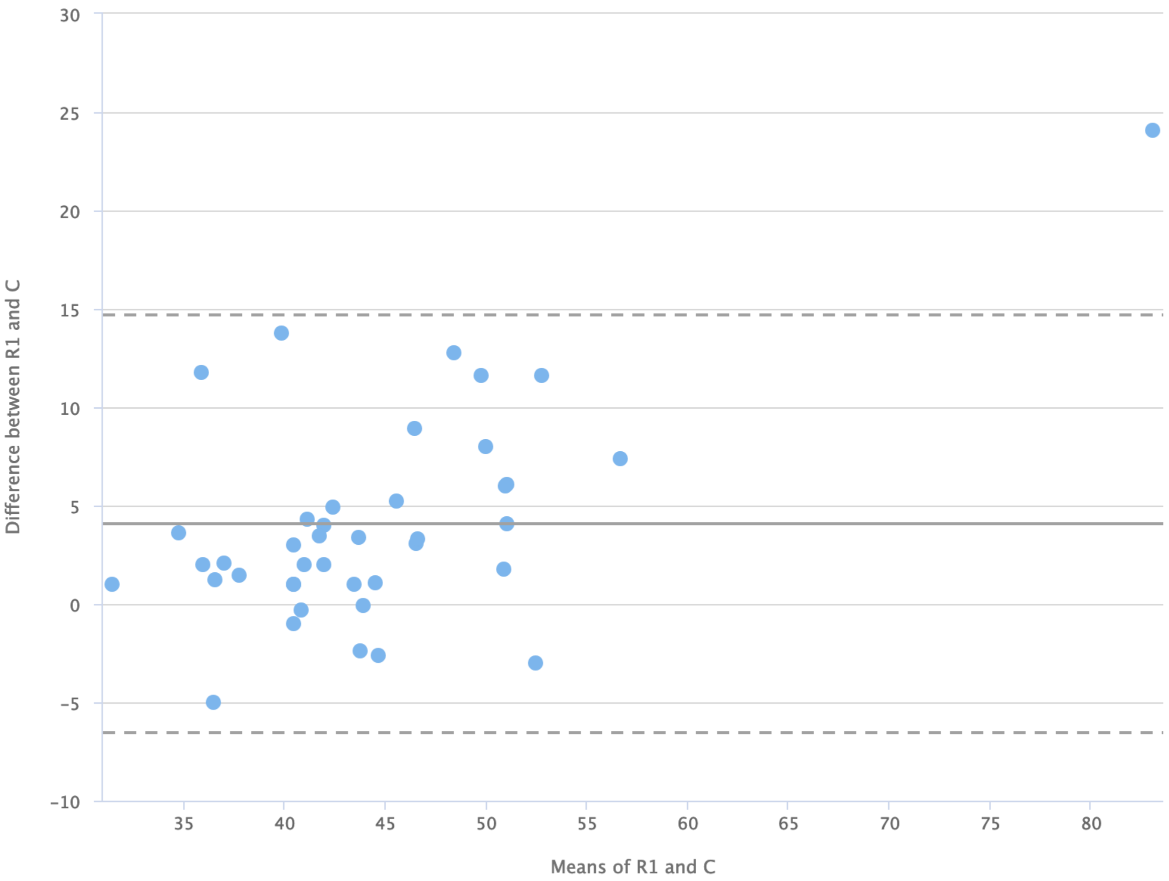

Figure S14. Bland Altman plot for the agreement between radiologist 1 and cardiologist for RA.

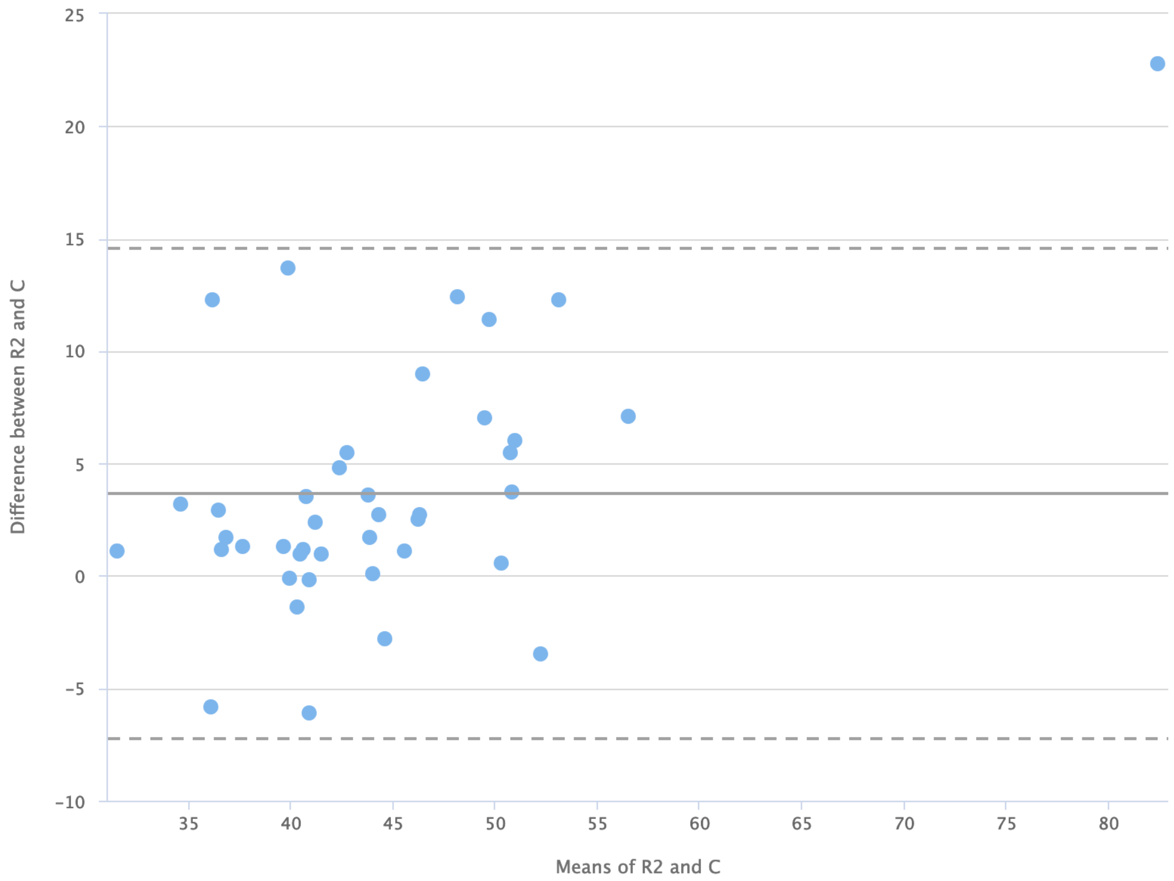

Figure S15. Bland Altman plot for the agreement between radiologist 2 and cardiologist for RA.

Table S1. Passing-Bablok Regression Analysis Results

	R1-R2			R1-C			R2-C		
	Estimation	Lower limit	Upper limit	Estimation	Lower limit	Upper limit	Estimation	Lower limit	Upper limit
**LVEDD**									
Intercept	-2.26	-8.52	4.23	2.04	-16.26	12.91	3.88	-10.21	18.20
Slope	1.02	0.88	1.15	0.92	0.70	1.30	0.93	0.62	1.22
**LVESD**									
Intercept	-1.36	-3.90	0.20	3.06	-5.23	9.02	5.89	-1.65	10.05
Slope	1.01	0.97	1.07	0.88	0.71	1.14	0.84	0.71	1.079
**LAPP**									
Intercept	-1.43	-3.50	0.20	6.14	-1.53	12.14	8.39	-0.02	14.23
Slope	1.04	0.99	1.09	0.85	0.68	1.11	0.79	0.62	1.03
**RV**									
Intercept	-2.25	-5.29	1.47	-1.49	-15.27	7.14	0.33	-12.00	6.49
Slope	1.04	0.96	1.10	0.98	0.76	1.31	0.95	0.800	1.25
**RA**									
Intercept	-0.11	-2.07	1.42	7.02	-2.50	13.70	7.40	-2.38	16.39
Slope	0.99	0.96	1.03	0.76	0.60	1.00	0.75	0.57	1

Abbreviations: R1: Radiologist 1, R2: Radiologist 2 C: Cardiologist, LVED: Left ventricular end diastolic diameter, LVES: Left ventricular end-systolic end, LAAP: Left atrial anteroposterior, RV: Right ventricle, RA: Right atrium.
